# The Process of Analyzing Data is the Emergent Feature of Data Science

**DOI:** 10.3389/fgene.2016.00012

**Published:** 2016-02-09

**Authors:** Frank Emmert-Streib, Salissou Moutari, Matthias Dehmer

**Affiliations:** ^1^Computational Medicine and Statistical Learning Laboratory, Department of Signal Processing, Tampere University of TechnologyTampere, Finland; ^2^Centre for Statistical Science and Operational Research, School of Mathematics and Physics, Queen's University BelfastBelfast, UK; ^3^Department of Computer Science, Institute for Theoretical Informatics, Mathematics and Operations Research, Universität der Bundeswehr MünchenNeubiberg, Germany

**Keywords:** data science, computational biology, statistics, big data, high-throughput data

In recent years the term “data science” gained considerable attention worldwide. In a *A Very Short History Of Data Science* by Press (2013), the first appearance of the term is ascribed to Peter Naur in 1974 (Concise Survey of Computer Methods). Regardless who used the term first and in what context it has been used, we think that data science is a good term to indicate that *data* are the focus of scientific research. This is in analogy to computer science, where the first department of computer science in the USA had been established in 1962 at Purdue University, at a time when the first electronic computers became available and it was still not clear enough what computers can do, one created therefore a new field where the *computer* was the focus of the study. In this paper, we want to address a couple of questions in order to demystify the meaning and the goals of data science in general.

The first question that comes to mind when hearing there is a new field is, what makes such existing field different from others? For this purpose Drew Conway created the data science Venn diagram (Conway, 2003) that is helpful in this discussion. In Figure 1, we show a modified version of the original diagram as an Efron-triangle (Efron, 2003), which includes metric information. The important point to realize is that data science is not an entirely new field in the sense that it deals with problems outside of any other field. Instead, the new contribution is its composition, consisting of at least three major fields, or dimensions, namely (1) domain knowledge, (2) statistics/mathematics, and (3) computer science. Here “domain knowledge” corresponds to a field that generates the data, e.g., biology, economics, finance, medicine, sociology, psychology etc. The position of a particular field in Figure 1, respectively the distances to the three corners of the triangle, i.e., (*d*_1_, *d*_2_, *d*_3_), provide information about the contribution of the three major fields, which can be seen as proportions or weights (see the examples in Figure 1). Overall, data science emerges at the intersection of these three fields whereas the term *emerges* is important because there is more than just “adding” the three parts together.

**Figure 1:**
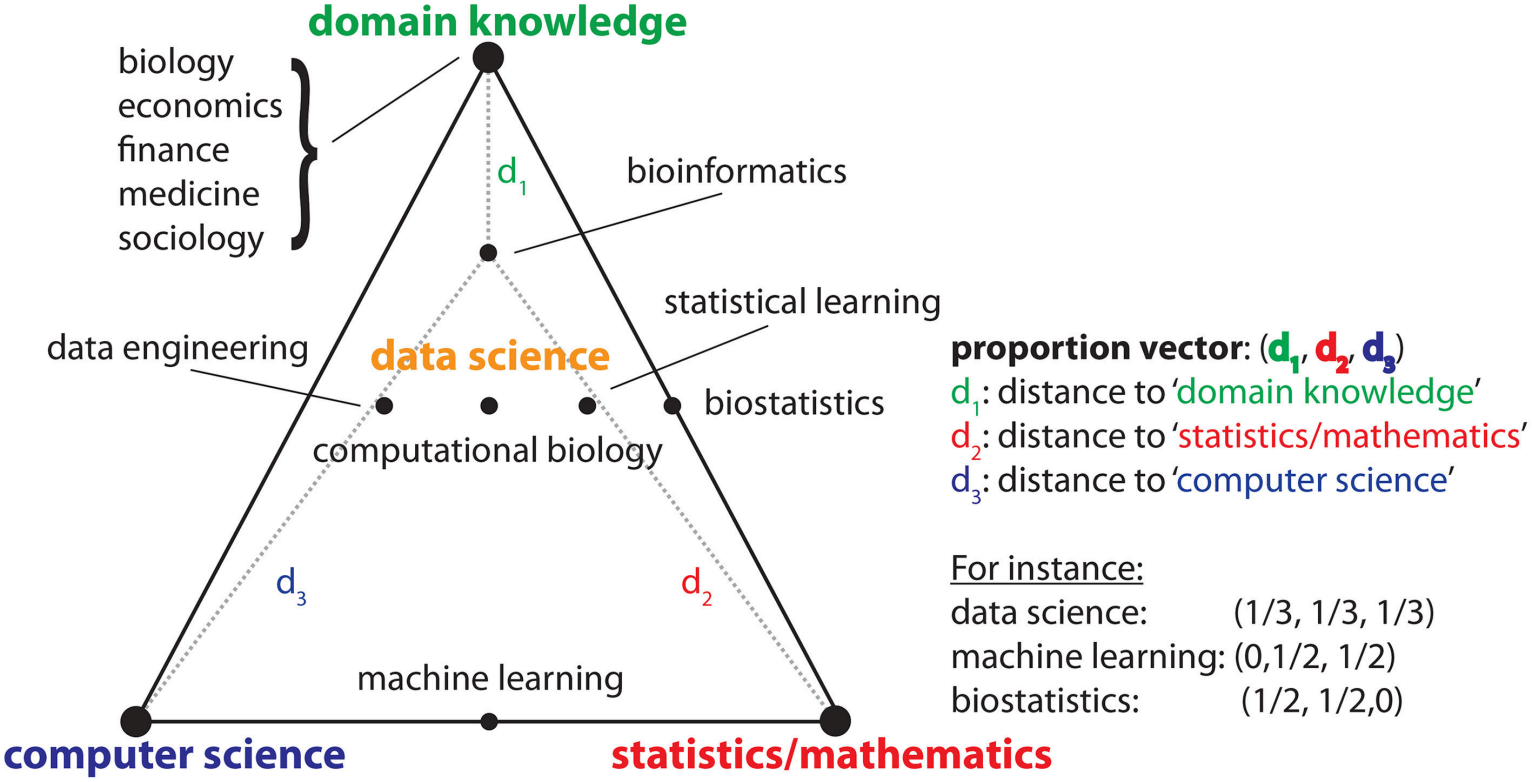
**Schematic visualization of the constituting parts of data science and other disciplines in terms of the involvement of (1) domain knowledge, (2) statistics/mathematics, and (3) computer science**.

Before we come back to the discussion of the emergent aspect of data science let us give some specific examples for existing fields in the light of data science in particular application domains. Scientific fields with a long history analyzing data by means of statistical methods are biostatistics and applied statistics. However, the computer science component in these fields is not very noticeable despite the fact that also algorithmic methods are used, e.g., via software package like SPSS (Statistical Package for the Social Sciences) or SAS (Statistical Analysis System). However, such software packages are not fully flexible programming languages but provide only a rather limited set of statistical and visualization functions that can be applied to a data set, usually, via a graphical user interface (GUI). Also, the preprocessing of the data sets themselves is difficult within the provided capabilities of such packages because of the lack of basic data manipulation functions. A more recent field that evolved from elements in biostatistics and bioinformatics is computational biology. Depending on its definition, which differs somewhat between the US and Europe, in general, computational biology is an example for a data science with a specific focus on biological and biomedical data. This is especially true since the completion of the Human Genome Project has led to a series of new and affordable high-throughput technologies that allow the generating of a variety of different types of 'omics data (Quackenbush, 2011). Also, initiatives like The Cancer Genome Atlas (TCGA) (The Cancer Genome Atlas Research Network, 2008) making the results of such large-scale experiments publicly available in the form of databases contributed considerably to the establishment of computational biology as a data science, because without such data availability, there would not be data science at all.

Maybe the best non-biological example of a problem that is purely based on data is the Stock Market. Here the prices of shares and stocks are continuously determined by the demand and supply level of electronic transactions enabled by the different markets. For instance, the goal of day traders is to recognize stable patterns within ordinary stochastic variations of price movements to forecast future prices reliably. Due to the fact that a typical time scale of a buying and selling cycle is within (a fraction of) a trading day these prices are usually beyond real value differences, e.g., of productivity changes of the companies themselves corresponding to the traded shares, but are more an expression of the expectations of the shareholders. For this reason, economic knowledge about balance sheets, income statements and cash flow statements are not sufficient for making informed trading decisions because the chart patterns need to be analyzed themselves by means of statistical algorithms.

From Figure 1, one might get the feeling that a data scientist is expected to have every skill of all three major fields. This is not true, but merely an impression of the two-dimensional projection of a multidimensional problem. For instance, it would not be expected from a data scientist to prove theorems about the convergence of a learning algorithm (Vapnik, 1995). This would be within the skill set of a (mathematical) statistician or a statistical learning theoretician. Also, conducting wet lab experiments leading to the generation of the data itself is outside the skill set. That means a data scientist needs to have interdisciplinary skills from a couple of different disciplines but does not need to possess a complete skill set from all of these fields. For this reason, these core disciplines do not become redundant or obsolete, but will still make important contributions beyond data science.

Importantly, there is an emergent component to data science that cannot be explained by the linear summation of its constituting elements described above. In our opinion this emergent component is the dynamical aspect that makes every data analysis a *process*. Others have named this data analysis process the data analysis cycle (Hardin et al., 2015). This includes the overall assessment of the problem, experimental design, data acquisition, data cleaning, data transformation, modeling and interpretation, prediction and the performance of an exploratory as well as confirmatory analysis. Especially the exploratory data analysis part (Tukey, 1977) makes it clear that there is an interaction process between the data analyst and the data under investigation, which follows a systematic approach, but is strongly influenced by the feedback of previous analysis steps making a static description from the outset usually impossible. Metaphorically, the result of a data analysis process is like a cocktail having a taste that is beyond its constituting ingredients. The process character of the analysis is also an expression of the fact that, typically, there is not just one method that allows answering a complex, domain specific question but the consecutive application of multiple methods allows achieving this. From this perspective it becomes also clear why statistical inference is more than the mere understanding of the technicalities of one method but that the output of one method forms the input of another method which is requires the sequential understanding of decision processes.

From the above description, a layman may think that statistics is data science, because the above elements can also be found in statistics. However, this is not true. It is more what statistics *could* be! Interestingly, we think it is fair to say that some (if not all) of the founders of statistics, e.g., Fisher or Pearson can be considered as data scientists because they (1) analyzed and showed genuine interest in a large number of different data types and their underlying phenomena, (2) possessed mathematical skills to develop new methods for their analysis, and (3) gathered large amounts of data and crunched numbers (by human labor). From this perspective one may wonder how statistics, that started out as data science, could end at a different point? Obviously, there had to be a driving force during the maturation of the field that prevented statistics from continuing along its original principles. We don't think this is due to the lack of creativity or insight of statisticians that didn't recognize this deviation, instead, we think that the institutionalization of statistics or science in general, e.g., by the formation of departments of statistics and their bureaucratic management as well as an era of slow progression in the development of data generation technologies, e.g., comparing the periods 1950–1970 with 2000-present, are major sources for this development. Given that, naturally, the beginning of every scientific discipline is not only indicated by the lack of a well defined description of the field and its goals, but rather by a collection of people who share a common vision. Therefore, it is clear that a formalization of a field leads inevitably to a restriction in its scope in order to form clear boundaries to other disciplines. In addition, the increasing introduction of managemental structures in universities and departments is an accelerating factor that led to a further reduction in flexibility and tolerability of the variability in individual research interests. The latter is certainly true for every discipline, not just statistics.

More specific to statistics is the fact that the technological progress between the 1930s and 1980s was rather slow compared to the developments within the last 30 years. These periods of stasis may have given the impression that developing fixed sets of methods is sufficient to deal with all possible problems. This may also explain the wide spread usage of software packages like SPSS or SAS among statisticians despite their limitations of not being fully flexible programming languages (Turing machines Turing, 1936), offering exactly these fixed sets of methods. In combination with the adaptation of the curriculum toward teaching students the usage of such software packages instead of programming languages, causes nowadays problems since the world changed and every year appear new technologies that are accompanied by data types with new and challenging characteristics; not to mention the integration of such data sets. Overall, all of these developments made statistics more applied and also less flexible, opening in this way the door for a new field to fill the gap. This field is data science.

A contribution that should not be underestimated in making, e.g., computational biology a data science is the development of the statistical programming language R pioneered by Robert Gentleman and Ross Ihaka (Altschul et al., 2013) and the establishment of the package repository Bioconductor (Gentleman et al., 2004). In our opinion the key to success is the flexibility of R being particularly suited for a statistical data analysis, yet having all features of a multi-purpose language and its interface to integrate programs written in other major languages like C++ or Fortran. Also, the license free availability for all major operating systems, including Windows, Apple and Linux, makes R an enabler for all kinds of data related problems that is in our opinion currently without rivalry.

Interestingly, despite the fact that computational biology has currently all attributes that makes it a data science, the case of statistics teaches us that this does not have to be this way forever. A potential danger to the field is to spend too much effort on the development of complex and complicated algorithms when in fact a solution can be achieved by simple methods. Furthermore, the iterative improvement of methods that lead only to marginal improvements of results consumes large amounts of resources that distract from the original problem buried within given data sets. Last but not least, the increasing institutionalization of computational biology at universities and research centers may lead to a less flexible field as discussed above. All such influences need to be battled because otherwise, in a couple of years from now, people may wonder how computational biology could leave the trajectory from being a data science.

## Author contributions

FS conceived the study. FS, SM, and MD wrote the paper and approved the final version.

## Funding

MD thanks the Austrian Science Funds for supporting this work (project P26142). MD gratefully acknowledges financial support from the German Federal Ministry of Education and Research (BMBF) (project RiKoV, Grant No. 13N12304).

### Conflict of interest statement

The authors declare that the research was conducted in the absence of any commercial or financial relationships that could be construed as a potential conflict of interest.
